# Multi-AGV task scheduling and dynamic map path planning based on task pre-allocation

**DOI:** 10.1371/journal.pone.0352782

**Published:** 2026-07-24

**Authors:** Jie Gao, Weinan Xie, Haoya Liu, Junda Zhou, Liang Wang, Jieke Liang

**Affiliations:** 1 School of Electronic Information Engineering, Shandong University of Science and Technology, Qingdao, Shandong, China; 2 School of Mechanical and Electronic Engineering, Shandong University of Science and Technology, Qingdao, Shandong, China; Xidian University, CHINA

## Abstract

Multi-AGV (Automated Guided Vehicle) systems operating in complex warehouse environments equipped with movable containers encounter several challenges, including high system no-load rate, low task response efficiency, and imbalanced path utilization. To address these issues, we propose an integrated optimization approach for task scheduling and path planning. First, a task segmentation strategy is introduced to decompose complex tasks into long-distance transport and precision in-racking sub-tasks which are then allocated to heterogeneous AGV types for execution. Second, a task pre-allocation algorithm is designed to enable AGVs to participate in the subsequent task assignment prior to the completion of their current tasks, thereby reducing the system no-load rate. Third, a dynamic map path planning mechanism is developed, which incorporates a temporary path traffic control module and a localized Floyd update algorithm to achieve real-time path adjustment and effective path utilization optimization. Comparative experiments were conducted in a multi-zone warehouse simulation environment. The results demonstrate that the proposed approach can reduce the system no-load rate, mitigate path congestion, and enhance overall operational performance.

## Introduction

AGV (Automated Guided Vehicle) technology originated in the 1950s to address the excessive consumption of labor and resources in manufacturing through automated material handling [1]. With continuous advancements in sensors, navigation systems, and communication technologies, AGVs have been widely deployed in manufacturing [2], warehouse logistics [3], healthcare [4], and other domains. In intelligent warehouse systems, multi-AGV cooperation has become a key approach to improving operational efficiency and system automation. However, as warehouse environments become more complex and task demands more dynamic, the coordinated optimization of task scheduling and path planning remains a critical challenge.

From the perspective of system operation, task scheduling efficiency plays a fundamental role in determining overall performance. High no-load rates and delayed task responses can reduce system utilization and responsiveness. To address these issues, various studies have focused on optimizing task allocation strategies. Yang [5] incorporated AGV empty travel distance into optimization objectives, while Ventura [6] proposed a genetic algorithm to reduce response time in warehouse systems. Zhu [7] introduced task allocation balancing and priority mechanisms to improve AGV utilization. Despite these efforts, most existing approaches require AGVs to complete their current tasks or return to designated waiting stations before receiving new assignments [8]. This leads to increased no-load rates and reduced scheduling continuity. In addition, the coordination of heterogeneous AGVs with different functional capabilities is often insufficiently considered [9], limiting system efficiency in complex operational scenarios.

Path planning and conflict management constitute another essential aspect of multi-AGV systems. Numerous studies have been conducted to improve path optimization and avoid collisions. Xu et al. [10] proposed a spatiotemporal simulated annealing algorithm to achieve global path optimization, while He et al. [11] combined the Dijkstra algorithm with time window constraints to generate collision-free paths. Zhuge et al. [12] modeled AGV scheduling as a weighted minimum path cover problem. Furthermore, conflict prediction and avoidance methods based on graph theory and spatiotemporal analysis have been widely investigated [13–15]. Although these approaches effectively improve path safety and feasibility, many of them assume relatively static environments and lack the ability to dynamically adjust paths under changing traffic conditions. As a result, issues such as path congestion and inefficient resource utilization may arise in large-scale systems.

With the increasing adoption of movable containers and flexible storage systems, warehouse environments are becoming highly dynamic. The frequent changes in obstacle positions make traditional path planning methods based on static maps less effective. Although some studies have attempted to incorporate dynamic environmental factors [16–17], most approaches rely on global map updates, which incur significant computational overhead and limit real-time responsiveness. This poses a challenge for achieving efficient and adaptive path planning in dynamic multi-AGV systems.

In summary, existing research still faces several limitations in supporting efficient and coordinated multi-AGV operations in complex warehouse environments: (1) insufficient consideration of heterogeneous AGV collaboration in task scheduling, leading to suboptimal resource utilization; (2) reliance on sequential task assignment mechanisms, resulting in high no-load rates and reduced scheduling efficiency; and (3) limited adaptability of path planning methods in dynamic environments with frequently changing map structures.

To address these challenges, this paper proposes an integrated optimization approach for task scheduling and dynamic map path planning based on task pre-allocation. The main contributions of this paper are summarized as follows.

(1) Task segmentation strategy: Complex tasks are decomposed into long-distance transportation and precision in-racking subtasks, which are allocated to heterogeneous AGVs for collaborative execution. This approach aligns task assignments with AGV functional differences, improving resource utilization.
(2) Task pre-allocation algorithm: This algorithm enables AGVs to participate in subsequent task assignment prior to completing their current tasks, eliminating the need to return to waiting stations. This reduces system no-load rates and improves scheduling continuity.
(3) Dynamic map path planning mechanism: A dynamic map-based planning framework is developed, integrating a temporary path traffic control module and a localized Floyd update algorithm. By restricting updates to affected regions, the proposed method reduces computational complexity and improves real-time responsiveness in dynamic environments.

The remainder of this paper is organized as follows. The Problem Formulation section presents the problem description and system model. The Floyd Algorithm section introduces the Floyd algorithm and its application in path planning. The System Design section describes the proposed task scheduling and dynamic path planning methods. The Simulation and Analysis section presents simulation results and performance analysis. The final section concludes the paper.

## Problem formulation

### Warehouse environment

**Fig 1:**
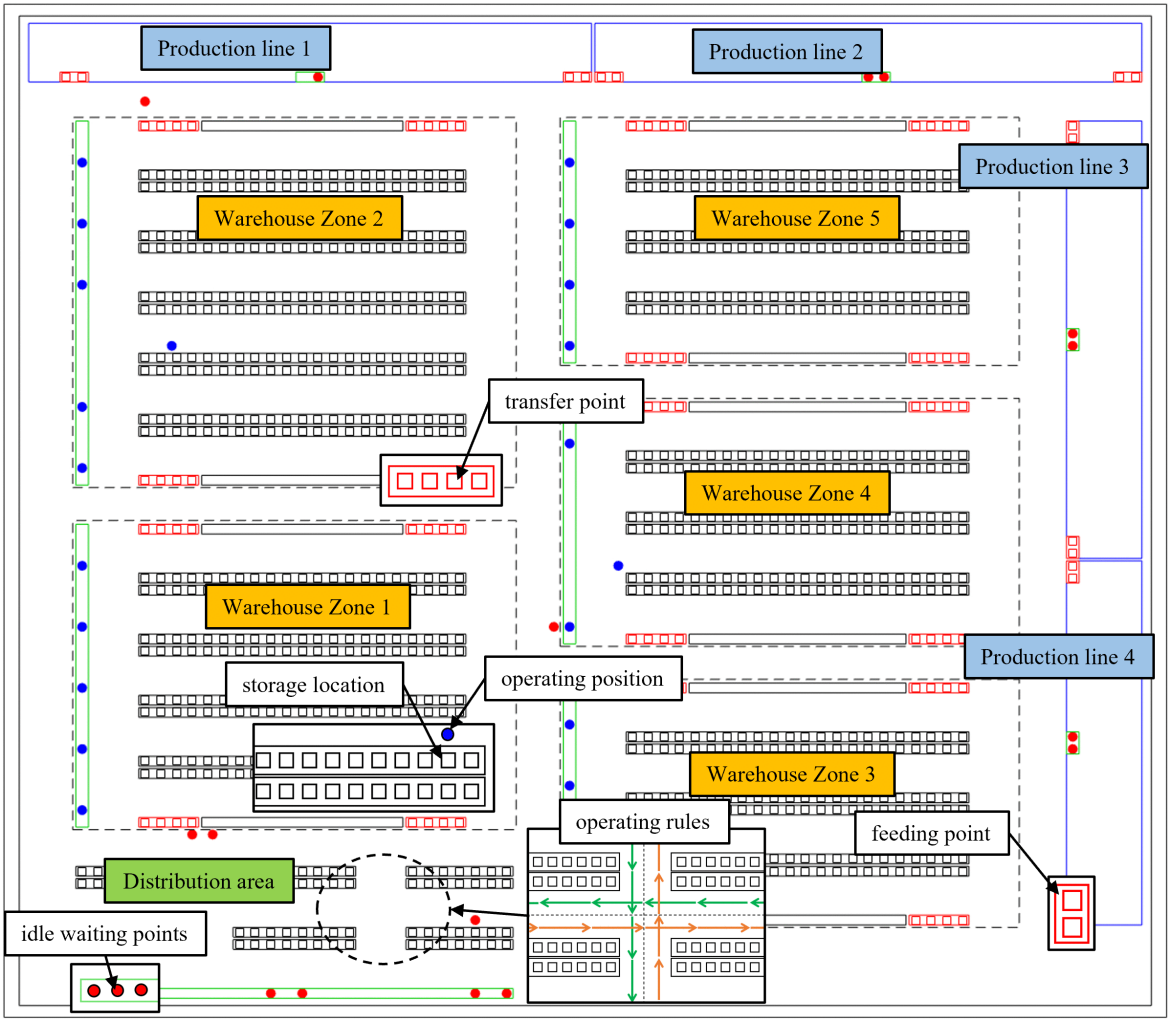
System layout.

This paper addresses the problem of task scheduling and path planning for multiple AGVs system in manufacturing logistics scenarios. A typical operational environment consisting of multiple zones is constructed in accordance with the production workshop environment of a certain factory. There are two types of AGVs with different functionalities to meet the requirements of handling tasks. The overall system layout, as illustrated in Fig 1, comprises a distribution area, multiple warehouse zones, and a series of production lines. The distribution area contains four 2 × 7 and two 2 × 8 storage sections, totaling 88 storage locations, which are designated for the temporary placement of materials pending warehousing. The distribution area comprises four 2 × 7 and two 2 × 8 storage sections, totaling 88 storage locations, which are designated for the temporary placement of materials pending warehousing. Warehouse Zone 1 consists of four 2 × 21 storage sections, providing a total of 168 storage locations. Warehouse Zone 2 consists of five 2 × 21 storage sections, providing a total of 210 storage locations. Warehouse Zones 3–5 each consist of three 2 × 22 storage sections, with 132 storage locations in each zone. Each of the five warehouse zones is configured with a 4 × 4 set of transfer points at its corners. Each of the four production lines is configured with a 2 × 2 set of feeding points at both ends. Additionally, waiting stations for AGVs are located below the distribution area, to the left of the warehouse zones, and at the midpoint of the production lines.

Each storage location and feeding point corresponds to one AGV operating point, while each transfer point corresponds to two AGV operating points. The number of AGVs and operating points allocated to each zone, determined in accordance with storage and production operational requirements, is presented in Table 1.

**Table 1:** Number of AGVs and operation points in each area.

	Distribution area	Warehouse Zone 1	Warehouse Zone 2	Warehouse Zones 3–5	Production line 1–4
**AGV number**	9	5	6	4	2
**Operation point number**	88	184	226	148	4

**Fig 2:**
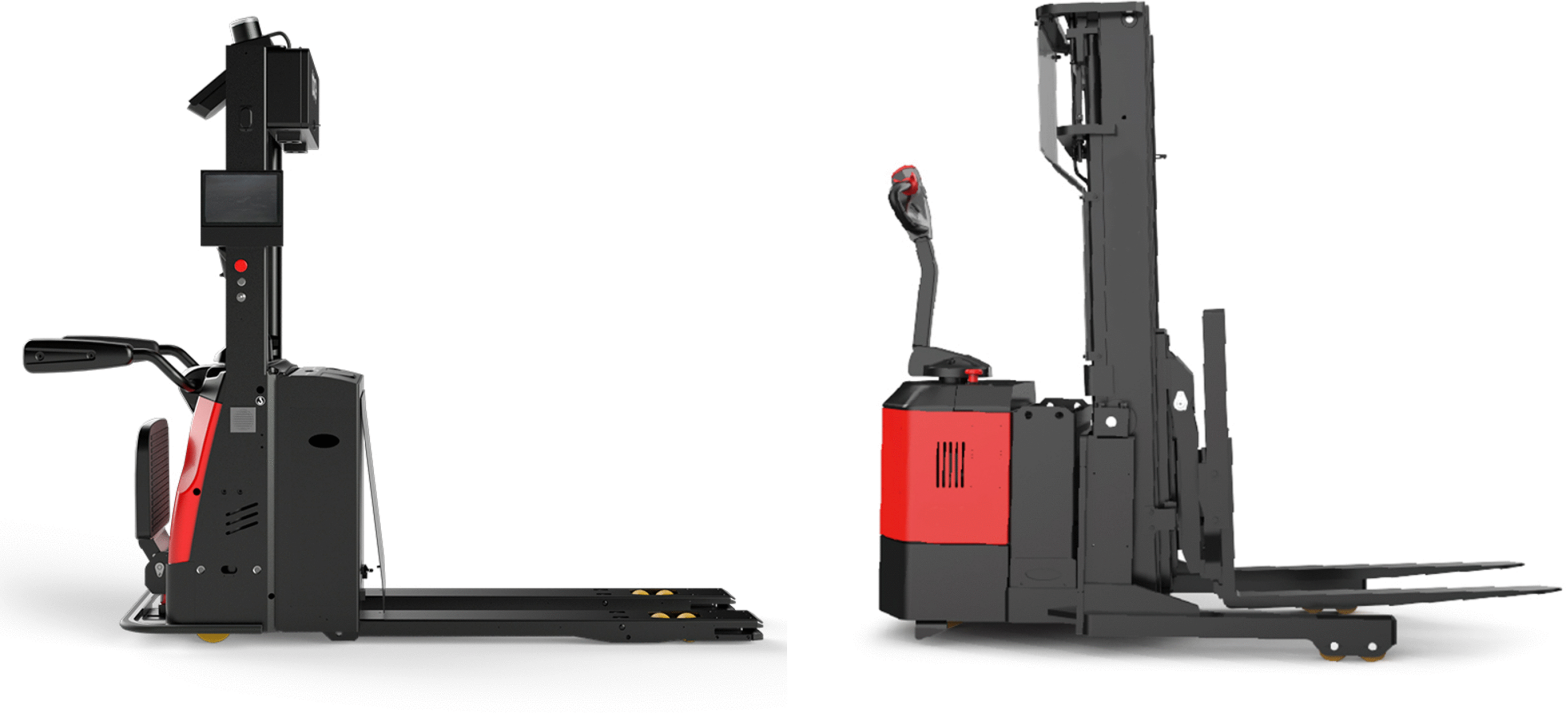
Two types of AGVs: (a) XP-type forklift; (b) XQE-type forklift.

In terms of AGV types, the system is equipped with two categories of AGVs to enable task specialization and collaborative operations. As illustrated in Fig 2(a), the XP-type forklift is designated for long-distance cross-zone transportation, with its primary function being to handle material transfers between the distribution area and warehouse zones, as well as between warehouse zones and production lines. By contrast, as depicted in Fig 2(b), the XQE-type forklift is dedicated to stacking operations within warehouse zones. Endowed with precise handling and stacking capabilities, this type of forklift operates exclusively inside each warehouse zone and does not participate directly in cross-zone scheduling.

**Fig 3:**
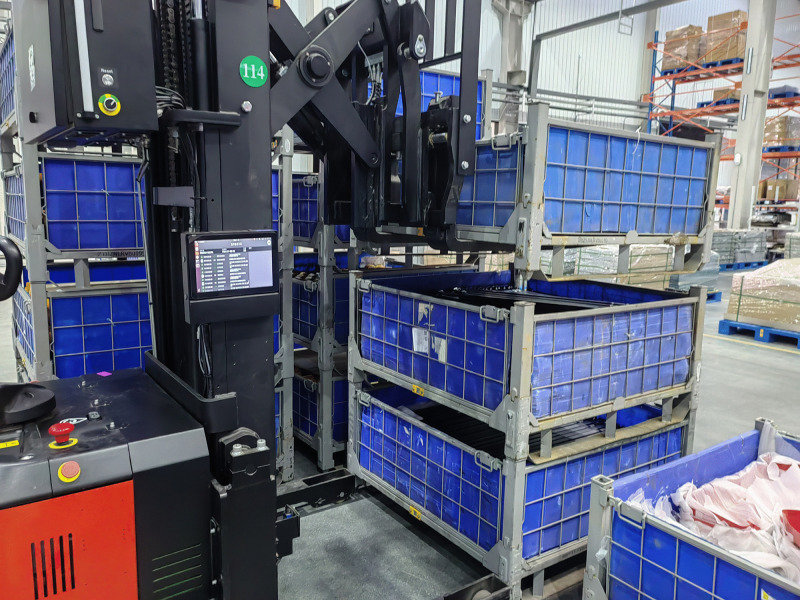
Movable containers.

As illustrated in Fig 3, the movable containers serve not only as material loading cage but also as simple storage racks in this system. In the handling process, all AGVs take movable containers as the basic carriers to enable the orderly circulation of materials between different functional zones.

### Basic operating rules of the system

AGVs operate under unified traffic rules to ensure orderly and safe multi-vehicle coordination. Specifically, AGV lanes are designated as one-way roads, and all AGVs follow the rule of keeping to the right while traveling within these lanes. As illustrated in Fig 1, when traversing intersection nodes, AGVs execute maneuvers such as straight travel, right turns, or left turns based on their target directions. AGVs performing turning operations must complete their directional adjustments only after confirming that no traffic conflicts will arise.

Based on the differences in AGV types and their respective operating areas, this paper constructs six adjacency matrices to characterize the path topologies within their corresponding regions. Specifically, XP-type AGVs are deployed to connect the distribution area, warehouse zones, and production lines, with their travel paths uniformly modeled as a single adjacency matrix denoted as $G_{xp}$. By contrast, XQE-type AGVs are restricted to exclusive operation within warehouse zones. The system is partitioned into five independent storage regions, each of which is modeled using a dedicated adjacency matrix denoted as $G_{xqe}^{k}$, where k=1,2,3,4,5. Each adjacency matrix G(i,j) characterizes the travel weight from node i to node j, subject to the following rules:


$$\begin{array}{c} 𝐆\left(i,j\right)=\left\{ \begin{array}{c} 0,\;if\;i=j\\ w_{ij},\;\;if\text{\textasciitilde{}}i\neq j\text{\textasciitilde{}}and\text{\textasciitilde{}}nodes\text{\textasciitilde{}}i\text{\textasciitilde{}}and\text{\textasciitilde{}}j\text{\textasciitilde{}}are\text{\textasciitilde{}}adjacent\\ \infty ,\text{\textasciitilde{}\textasciitilde{}\textasciitilde{}}if\text{\textasciitilde{}}nodes\text{\textasciitilde{}}i\text{\textasciitilde{}}and\text{\textasciitilde{}}j\text{\textasciitilde{}}are\text{\textasciitilde{}}not\text{\textasciitilde{}}adjacent \end{array}\right. \end{array}$$

**Fig 4:**
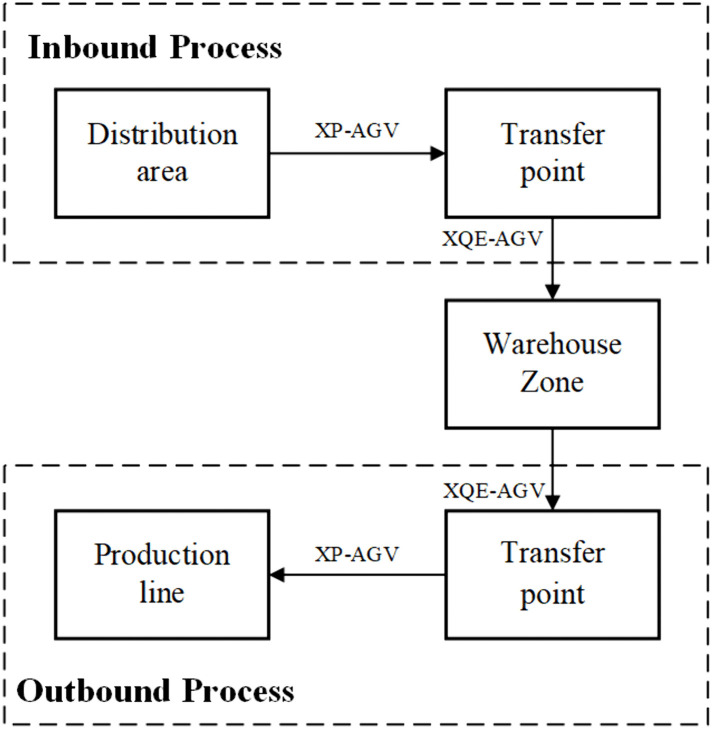
Schematic of the inbound and outbound processes.

During the operation of the warehouse system, the scheduling system sequentially dispatches transportation tasks according to the operational coordination and material flows between the upstream distribution area and the downstream production lines. There are two kinds of tasks: inbound and outbound operations. In inbound operations, the XP-type forklift in the distribution area first transports the materials from the distribution area to the designated transfer point of the target warehouse zone. The XQE-type forklift in that warehouse zone then retrieves the materials from the transfer point and stacks them at the designated location within the warehouse. The outbound process is the reverse: the XQE-type forklift removes the target materials from the stacking area, delivers them to the warehouse zone’s transfer point, and then the XP-type forklift from the production line transfers them to the corresponding feeding point of the production line to complete the loading. Once a task is dispatched, it must be assigned to an suitable AGV, and a corresponding execution path must be generated. A schematic diagram of the inbound and outbound processes is illustrated in Fig 4.

To enable efficient collaboration between the two AGV types, this paper introduces a task segmentation strategy. This strategy allows the system to allocate tasks based on the functional characteristics of each AGV type, thereby rationalizing the assignment of task routes and operational zones. During task segmentation process, the system must first identify optimal handover locations, namely the coordinates of the transfer points. As indicated in the system map, each warehouse zone is configured with 16 transfer points. Each transfer point is equipped with one dedicated working position for an XP-type AGV and one for an XQE-type AGV to facilitate cargo handover operations. To unify the spatial and status management of transfer points, a matrix is defined for Warehouse Zone n to record the coordinates of its two working positions and their occupancy status, as shown below:


$$\begin{array}{c} T_{n}=\left[\begin{array}{ccccc} x_{\text{1}}^{p} & y_{\text{1}}^{p} & x_{\text{1}}^{q} & y_{\text{1}}^{q} & o_{\text{1}}\\ x_{\text{2}}^{p} & y_{\text{2}}^{p} & x_{\text{2}}^{q} & y_{\text{2}}^{q} & o_{\text{2}}\\ \vdots & \vdots & \vdots & \vdots & \vdots \\ x_{16}^{p} & y_{16}^{p} & x_{16}^{q} & y_{16}^{q} & o_{16} \end{array}\right] \end{array}$$ (1)

Each row of the matrix represents the information of one transfer point, where $\left(x_{\alpha }^{p},y_{\alpha }^{p}\right)$ and $\left(x_{\alpha }^{q},y_{\alpha }^{q}\right)$ denote the working position coordinates of the XQE- and XP-type AGVs at the transfer point α, respectively. $o_{\alpha }\in \left\{0,1\right\}$ indicates the occupancy status, with $o_{\alpha }=1$ meaning the point is occupied.

Taking the inbound task as an example, when the system receives a transportation task, it defines the starting point $s=\left(x_{s},y_{s}\right)$, the end point $e=\left(x_{e},y_{e}\right)$, and the coordinates of the transfer point *n* as $p_{\alpha }=\left[(x_{\alpha }^{p}+x_{\alpha }^{q})/2,(y_{\alpha }^{p}+y_{\alpha }^{q})/2\right]$. The goal is to find the index number $\alpha^{*}\in \left\{1,2,3..\mathrm{.16}\right\}$ of the transfer point that satisfies the condition below:


$$\begin{array}{c} \alpha^{*}=arg\underset{\alpha \in Z}{min}\left(\right||p_{\alpha }-s|{|}_{\text{1}}+\left|\right|p_{\alpha }-e\left|{|}_{\text{1}}\right) \end{array}$$ (2)

where $𝐙=\{i\in \{1,2,3,...,16\}|o_{i}=0\text{\textbackslash{}hspace\{0.5em}\}\}$. After segmentation, the pre-task information $m_{f}$ and post-task information $m_{b}$ are expressed as follows:


$$\begin{array}{c} m_{f}=\left[\begin{array}{cc} x_{s} & y_{s}\\ x_{\alpha^{*}}^{p} & y_{\alpha^{*}}^{p} \end{array}\right]\text{\textbackslash{}hspace\{0.5em}\}m_{b}=\left[\begin{array}{cc} x_{\alpha^{*}}^{q} & y_{\alpha^{*}}^{q}\\ x_{e} & y_{e} \end{array}\right] \end{array}$$ (3)

where $\left(x_{s},y_{s}\right)$ is the starting point and $\left(x_{\alpha^{*}}^{p},y_{\alpha^{*}}^{p}\right)$ is the end point of the pre-task, while $\left(x_{\alpha^{*}}^{q},y_{\alpha^{*}}^{q}\right)$ is the starting point and $\left(x_{e},y_{e}\right)$ is the end point of the post-task. This task segmentation strategy provides a basis for subsequent task path segmentation planning and resource allocation.

## Floyd algorithm

In conventional AGV path planning, the Dijkstra algorithm is a widely used single-source shortest path method [18]. Starting from the initial position, it incrementally expands outward on the map, at each step selecting the path node closest to the source as the basis for further exploration, repeating until the destination is included in the path [19]. The A* algorithm adopts a greedy strategy to find the shortest path [20], introducing a heuristic function to evaluate the desirability of candidate paths between nodes, enabling it to obtain an optimal solution within a relatively short time. However, for maps with complex paths and large numbers of nodes, both Dijkstra and A* often incur excessive computational overhead, which hinders their ability to determine the optimal route efficiently.

In this paper, the Floyd algorithm is adopted, which exhibits higher efficiency than Dijkstra’s algorithm and the A* algorithm in solving the all-pairs shortest path problem [21]. The Floyd algorithm is a dynamic programming method for computing the shortest paths between all pairs of vertices in a map. During path planning, it computes routes directly by reading distances between two points from the adjacency matrix. Its core idea is to iteratively traverse all vertices in the weighted map and update the estimated shortest distances between node pairs. The formula is as follows:


$$\begin{array}{c} 𝐃\left(i,j\right)=min\left\{𝐃\left(i,j\right),𝐃\left(i,k)+𝐃(k,j\right)\right\}(1\leq k\leq n,k\neq i\neq j) \end{array}$$ (4)

$$\begin{array}{c} 𝐏\left(i,j\right)=\left\{ \begin{array}{c} i,\text{\textbackslash{}hspace\{0.5em}\}𝐃\left(i,j\right)\leq 𝐃\left(i,k\right)+𝐃\left(k,j\right)\\ k,\text{\textbackslash{}hspace\{0.5em}\}𝐃\left(i,j\right)>𝐃\left(i,k\right)+𝐃\left(k,j\right) \end{array}\right.(1\leq k\leq n,k\neq i\neq j) \end{array}$$ (5)

1) **Initialization:**

Create an n×n adjacency matrix ***D***, where ***D***(*i*, *j*) represents the direct distance from node *i* to node *j*. If no direct connection exists, the distance is set to infinity. A path matrix ***P*** of the same size is also created, where ***P***(*i*, *j*) records the path information, for unconnected nodes, the value is set −1; for connected nodes, the value is set to the row index.

2) **Iterative update:**

For each node pair (*i*, *j*), use *k* as an intermediate node. If D(i,k)+D(k,j)<D(i,j), update D(i,j) with this smaller value and set P(i,j)=k, indicating that the shortest path from *i* to *j* passes through *k*.

3) **Traversal:**

Use *k* to iterate over all *n* vertices in the weighted map. After *n* iterations, the values in the distance matrix ***D*** will represent the shortest path lengths between all pairs of vertices, while retrieving the path only requires arranging the nodes from the path matrix ***P*** in reverse order.

Given that warehouse layouts only undergo localized modifications with a stable overall environment, a fast-computing algorithm is required to match these static characteristics, reducing computational overhead and improving system efficiency. As a classical graph-based algorithm, the Floyd algorithm can derive all-pairs shortest paths from the adjacency matrix in one execution, achieving both computational efficiency and global optimality. Thus, it is adopted in this paper as the fundamental path planning method for multi-AGV warehouse systems.

## System design

In practical warehouse environments, when AGVs transport movable container, the failure of the path-planning process to promptly adapt to dynamic environmental changes can lead to unnecessary waiting and detours, thereby reducing overall handling efficiency. Conventional scheduling strategies often assign a new task only after the current one is completed, which increases the AGV’s empty travel rate and limits resource utilization efficiency.

To address these issues, this paper integrates two approaches: a task pre-allocation algorithm and a dynamic map path planning mechanism. The former matches subsequent tasks prior to the completion of an AGV’s current task, reducing the frequency of empty dispatches and enabling coordinated optimization of route continuity and scheduling foresight. The latter dynamically identifies non-stacking areas and temporarily opens corresponding paths, and efficiently updating route information using an improved localized Floyd algorithm, thereby enhancing the system’s environmental adaptability.

### Task pre-allocation

In multi-AGV warehouse systems, conventional scheduling strategies typically assign the next task only after an AGV has completed its current task and returns to the waiting stations, which impairs the system’s response efficiency and scheduling continuity. This paper proposes a task pre-allocation algorithm that initiates the allocation process when an AGV is approaching the completion of its current task. The scheduling system selects the optimal pending task based on the spatial coordinates of the current task’s terminus, enabling task pre-allocation and seamless path concatenation for transportation tasks. This approach effectively reduces the system no-load rate, shortens task waiting time, and enhances the overall system efficiency.

To enable task pre-allocation, the system must quantitatively evaluate the response efficiency of each available AGV at the moment a transport order is issued. This paper adopts a time-cost–based evaluation strategy, via which the scheduling system estimates the total time required for an AGV to respond to a new task based on its current operating state. Considering different operational states of AGVs, this paper classifies their status into three categories and develops corresponding computational models for time-cost evaluation. The specific algorithm is presented as follows:

(1) If AGV is docked at the standby station when a transport order is released, the time cost for responding to the task equals the travel time from the standby station to the task’s starting point. The calculation formula is as follows:

$$\begin{array}{c} t_{\mu \omega }=\frac{D\left(o_{\mu },s_{\omega }\right)}{v_{\mu }} \end{array}$$ (6)

where ***D*** is the shortest distance matrix obtained by Floyd algorithm, $o_{\mu }$ is the coordinate of the standby station of AGV μ, $s_{\omega }$ is the coordinate of the starting point of transport order ω, and $v_{\mu }$ is the travel speed of AGV μ.

(2) If AGV μ is traveling back to its standby station without load when the transport order ω arrived, the time cost $t_{\mu \omega }$ equals the travel time from its current position $p_{\mu }$ to the starting point of the task. The calculation formula is as follows:

$$\begin{array}{c} t_{\mu \omega }=\frac{D\left(p_{\mu },s_{\omega }\right)}{v_{\mu }} \end{array}$$ (7)

(3) If AGV μ is executing an assigned transport order when the transport order ω arrived, the time cost $t_{\mu \omega }$ equals the total time required to complete all current tasks plus the travel time from the end point of the last current task to the starting point of task ω. The calculation formula is as follows:

$$\begin{array}{c} t_{\mu \omega }=\sum_{m=k_{\mu }}^{n_{\mu }-1}\frac{D\left(p_{\mu }^{m},p_{\mu }^{m+1}\right)}{v_{\mu }}+\sum_{m=k_{\mu }}^{n_{\mu }}t_{wm}+\frac{D\left(e_{\omega -1},s_{\omega }\right)}{v_{\mu }} \end{array}$$ (8)

where $n_{\mu }$ is the total number of operating points in the current task list of AGV μ, $k_{\mu }$ is the number of operating points that AGV μ has arrived at, $p_{\mu }^{m}$ is the position coordinates of the operating point *m* in the task list, $t_{wm}$ is the operation time required for AGV μ at the operating point *m*, and $e_{\omega -1}$ is the end point coordinates of the task ω−1 in the task list.

After estimating the time costs of AGVs under various states, the system adopts these costs as task allocation metrics. The AGV with the minimum time cost is deemed the most suitable for executing the pending task at the current moment. During the task assignment, the task pre-allocation algorithm achieves path connectivity and resource screening, while incorporating AGVs in all operational states into the evaluation scope. This not only reduces empty-load redundancy but also improves scheduling continuity.

### Dynamic map path planning

**Fig 5:**
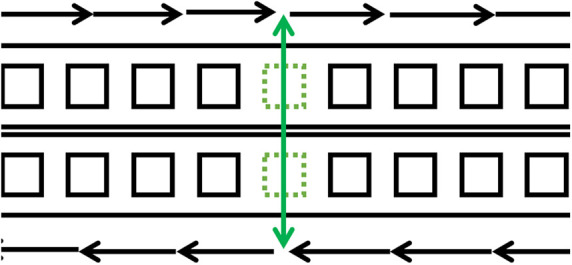
Formation of a temporary path.

To enhance the path adaptability of multi-AGV systems in warehouse environments, this paper develops a dynamic map path planning mechanism based on static path planning. This strategy addresses local path accessibility changes caused by variations in the stacking status of movable container. During the transport of movable container, areas that are initially inaccessible may become temporarily available as paths in the course of task execution, as illustrated in Fig 5.

If such areas can be dynamically marked as accessible temporary paths during the path-planning stage, path utilization can be improved, local traffic congestion alleviated, and conflict-induced waiting times reduced. Assuming the warehouse zone contains *n* movable containers, a location state matrix $S\in {\mathbb{R}}^{n\times 4}$ is defined to describe the operational status of each location, as follows:


$$\begin{array}{c} S=\left[\begin{array}{cccc} x_{\text{1}} & y_{\text{1}} & z_{\text{1}} & t_{\text{1}}\\ x_{\text{2}} & y_{\text{2}} & z_{\text{2}} & t_{\text{2}}\\ \vdots & \vdots & \vdots & \vdots \\ x_{n} & y_{n} & z_{n} & t_{n} \end{array}\right] \end{array}$$ (9)

Each row of the matrix represents a single location, where $\left(x_{i},y_{i}\right)$ denotes the Cartesian coordinate of node *i*, $z_{i}\in \left\{0,1\right\}$ is the placement status of movable containers, $z_{i}=0$ indicates that a movable container is placed at this location, and $t_{i}\in \left\{0,1\right\}$ is the temporary path flag. A value of $t_{i}=1$ indicates that the location can serve as a temporary path.

During operation, the system periodically inspects the states of vertically adjacent locations within the same column. If two consecutive locations are both unoccupied, they are deemed eligible to form a temporary path. The corresponding entries in the adjacency matrix between these nodes are then dynamically updated to reflect the new connectivity, enabling real-time updates of the path topology. Simultaneously, the system monitors all existing temporary paths. If any node along a temporary path becomes occupied by movable containers, the path is deemed invalid and is removed from the adjacency matrix. The detailed steps of the temporary path update algorithm are as follows:

**Algorithm temporary path update**

**Input:**   The location state matrix ***S***

       The adjacency matrix ***G***

**Output:** The updated adjacency matrix G'

      The distance matrix ***D***

      The path matrix ***P***

**1**  Initialize the adjacency matrix G'=G

**2**  **for** each i in 1 to n

**3**   Determine whether the path is passable

**4**    **if** $\left(y_{i}=y_{j}+\Delta y\right)\text{\textbackslash{}u2229}\left(x_{i}=x_{j}\right)\text{\textbackslash{}u2229}\left(z_{i}=z_{j}=0\right)$

**5**     Restore Path Weight

**6**     $G\text{'}\left(i,j\right)=G\text{'}\left(j,i\text{\textbackslash{}hspace\{0.5em}\;\}\right)=w_{t}$

**7**     $t_{i}=t_{j}=1$

**8**    **end**

**9**   Determine whether a temporary path exists

**10**   **if** $\left(y_{i}=y_{j}+\Delta y\right)\text{\textbackslash{}u2229}\left(x_{i}=x_{j}\right)\text{\textbackslash{}u2229}\left(t_{i}=t_{j}=1\right)$

**11**    Determine whether the temporary path is blocked

**12**    **if** $\left(z_{i}\neq 0\right)\text{\textbackslash{}hspace\{0.5em}\mathrm{\backslash u}222a\}\left(z_{j}\neq 0\right)$

**13**     Block the temporary path

**14**     G'(i,j)=G'(j,i\hspace{0.5em})=INF

**15**       $t_{i}=t_{j}=0$

**16**    **end**

**17**   **end**

**18**  **end**

**19** [D,P]=Floyd(G')

In the algorithm, Δy denotes the vertical distance between adjacent storage locations, $w_{t}$ is the weight assigned to the temporary path in the topological map, and Floyd(G') denotes the distance and path matrices derived from the Floyd algorithm implemented in accordance with Eq. (4) and Eq. (5).

**Fig 6:**
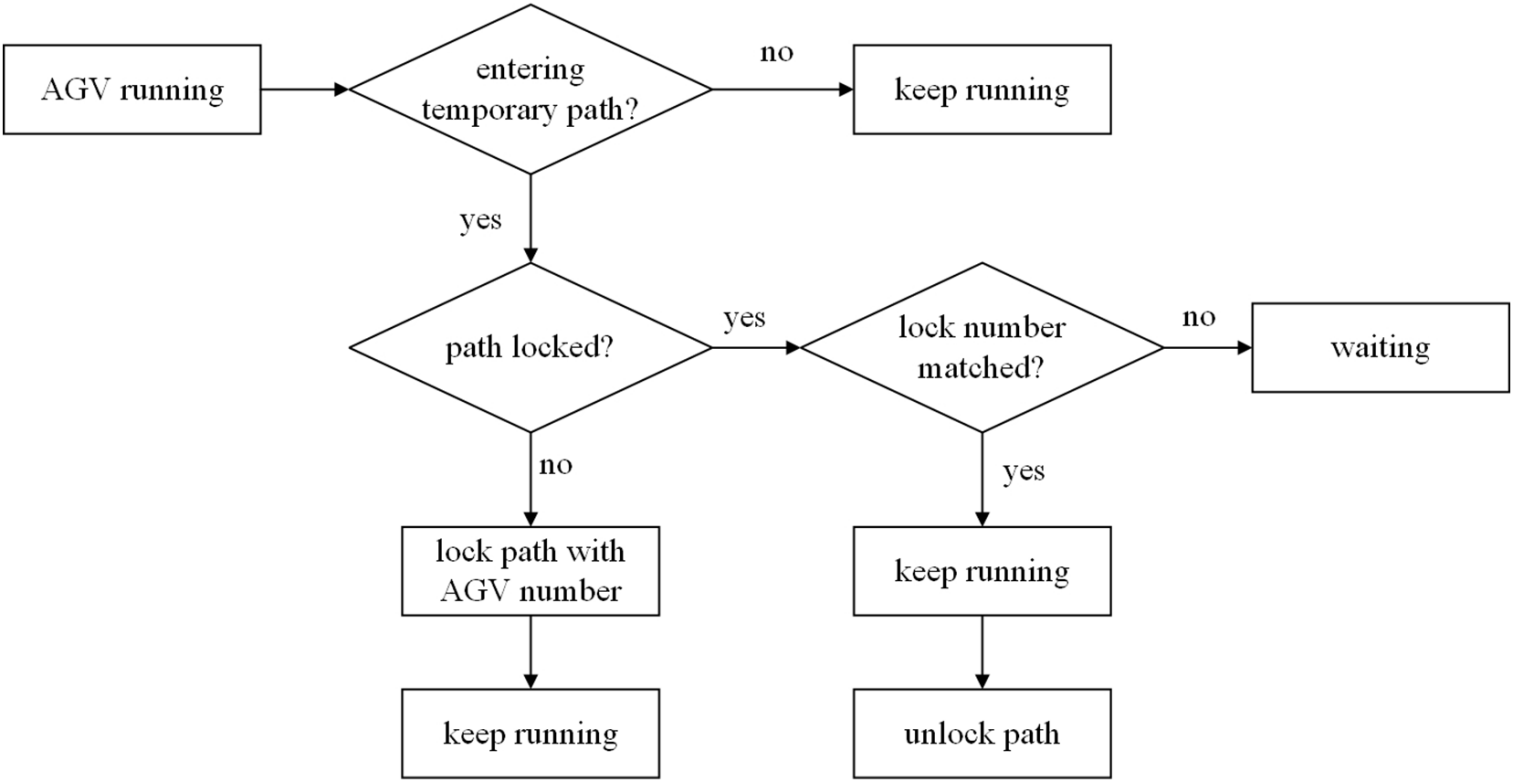
The decision-making process of the temporary-path traffic control module.

Since temporary paths are typically situated between storage positions in the warehouse and are bidirectional, simultaneous entry of multiple AGVs is prone to causing head-on conflicts, which impairs system efficiency and may even lead to deadlocks. To address this issue, a temporary-path traffic control module is developed to dynamically manage access permissions and coordinate scheduling. The module ensures that only one direction of travel is permitted for AGVs on a temporary path at any given time, thereby effectively preventing path conflicts and scheduling blockages arising from opposing traffic. The decision-making process of the traffic control module is illustrated in Fig 6.

Based on the control logic of path locking, identity verification, and path unlocking, the temporary path ensures directional exclusivity and AGV safety during operation. This mechanism effectively prevents head-on conflicts and path contention.

With the introduction of the temporary path, the system’s adjacency matrix ***G*** undergoes frequent updates due to dynamic changes in path status, which in turn affects the distance matrix ***D*** and path matrix ***P*** generated by the Floyd algorithm. Recomputing the full Floyd algorithm following each path change would incur considerable computational overhead and memory consumption, which impairs the system’s real-time responsiveness. To address this issue, an improved localized Floyd algorithm is proposed to update only the affected path regions in the adjacency matrix when a new temporary path emerges, thereby eliminating redundant computations across the entire graph. The algorithm is formulated as follows:


$$\begin{array}{c} D\text{'}\left(a,b\right)=\text{\textbackslash{}hspace\{0.5em}\;\}D\text{'}\left(b,a\right)=w_{temp} \end{array}$$ (10)

$$\begin{array}{c} D\text{'}\left(i,j\right)=min\left\{D\left(i,j\right),D\left(i,\text{a})+D(a,b)+D(b,j\right),D\left(i,\text{b})+D(b,a)+D(a,j\right)\right\} \end{array}$$ (11)

$$\begin{array}{c} 𝐏\text{'}\left(i,j\right)=\left\{ \begin{array}{c} 𝐏\left(i,j\right),\text{\textbackslash{}hspace\{0.5em}\}𝐃\left(i,j\right)\leq 𝐃\left(i,a\right)+𝐃\left(a,b\right)+𝐃\left(b,j\right)\text{\textbackslash{}hspace\{0.5em}\}and\text{\textbackslash{}hspace\{0.5em}\}𝐃\left(i,j\right)\leq 𝐃\left(i,b\right)+𝐃\left(b,a\right)+𝐃\left(a,j\right)\text{\textbackslash{}hspace\{0.5em}\}\\ 𝐏\left(a,j\right),\text{\textbackslash{}hspace\{0.5em}\}𝐃\left(i,j\right)>𝐃\left(i,a\right)+𝐃\left(a,b\right)+𝐃\left(b,j\right)\\ 𝐏\left(b,j\right),\text{\textbackslash{}hspace\{0.5em}\}𝐃\left(i,j\right)>𝐃\left(i,b\right)+𝐃\left(b,a\right)+𝐃\left(a,j\right) \end{array}\right. \end{array}$$ (12)

where D' and P' are the updated distance and path matrices, respectively; *i* is the starting node; *j* is the destination node; *n* is the number of nodes in the weighted map; *a* and *b* are the endpoints of the newly added temporary path in the topological map. The computation proceeds as follows:

1) **Local Initialization:**

During system operation, if a finite edge weight is assigned to two previously unconnected nodes *a* and *b* in the adjacency matrix ***G***—indicating the establishment of a direct passage—the corresponding elements in the distance matrix D' are set to the new cost $w_{temp}$, as shown in Eq. (10).

2) **Local Iteration:**

For each pair of nodes (*i*, *j*) in the map, nodes *a* and *b* are employed as intermediate nodes for comparison. If D(i,a)+D(a,b)+D(b,j) or D(i,b)+D(b,a)+D(a,j) is smaller than the current D(i,j), the distance matrix D' is updated as Eq. (11). Simultaneously, P'(i,j)is updated to P(a,j) or P(b,j), indicating that the shortest path from *i* to *j* passes through a→b or b→a, as shown in Eq. (12).

3) **Traversal:**

Since the impact of the updated path is locally confined, it is sufficient to traverse the other nodes in the topological map using a→b and b→a as intermediate paths to obtain the updated shortest distance matrix D' and path matrix P' after the temporary path is added.

Compared with the $O\left(n^{\text{3}}\right)$ time complexity of the traditional Floyd algorithm, the proposed method employs local updates to reduce the complexity to $O\left(n^{\text{2}}\right)$, decreasing computation time and memory overhead while enhancing system real-time performance.

### Multi-AGV task scheduling and dynamic map path planning algorithm based on task pre-allocation

**Fig 7:**
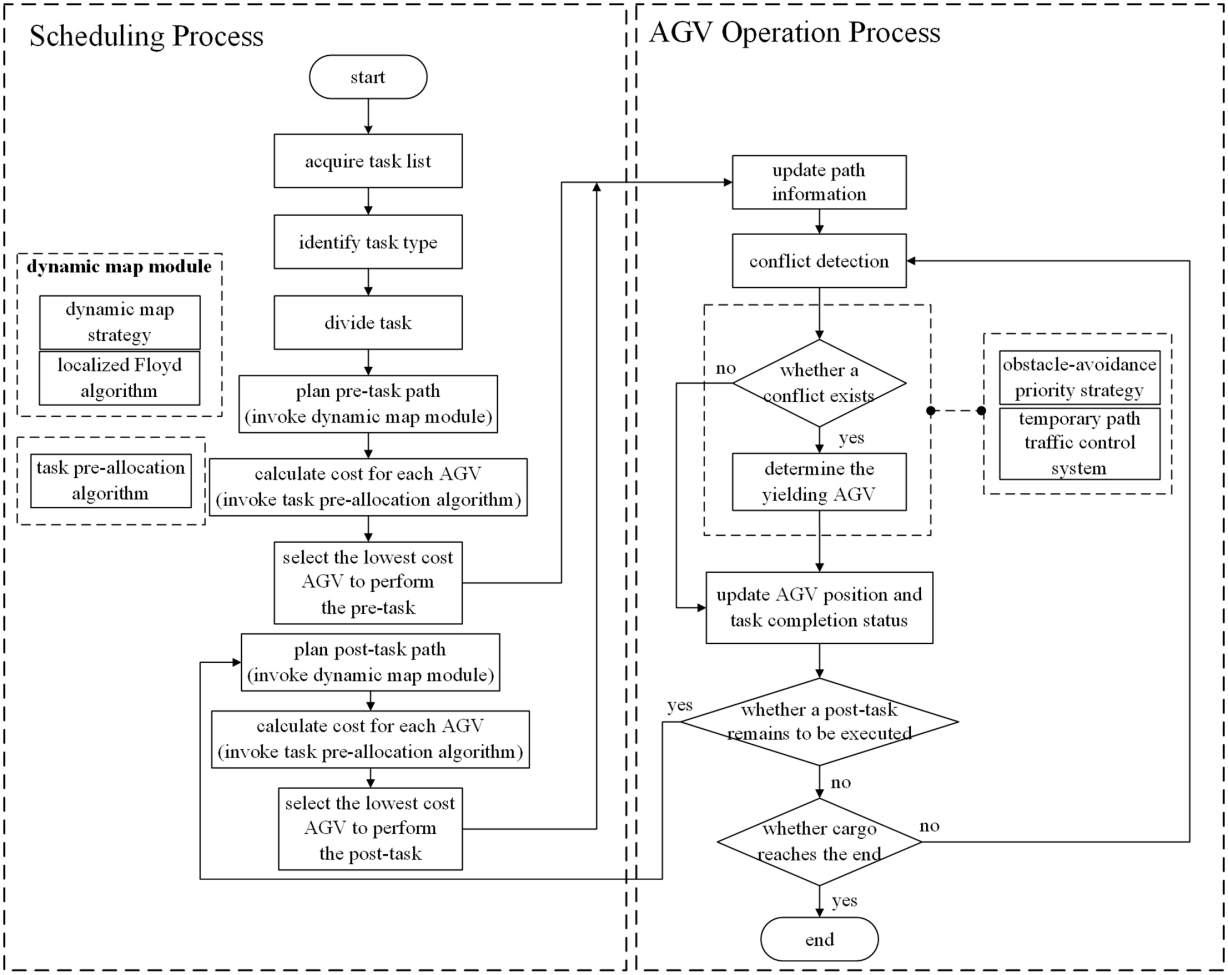
Flowchart of multi-AGV task scheduling and dynamic map path planning algorithm based on task pre-allocation.

The flowchart of the proposed multi-AGV task scheduling and path planning algorithm, which integrates the task pre-allocation strategy, dynamic map mechanism, and the improved localized Floyd algorithm, is illustrated in Fig 7.

The system consists of two components: the scheduling process and the AGV operation process. In the scheduling process, the system first acquires task list and identifies task types, then decomposes the tasks into subtasks to be executed by XP-type and XQE-type AGVs, respectively. It calculates the sum of Manhattan distances from each transfer point to the task’s start and end points, and selects the point with the minimum total distance as the handover location between the pre-task and post-task. Based on the real-time stacking status in the warehouse zone, the dynamic map module updates the adjacency matrix; subsequently, the localized Floyd algorithm efficiently refreshes the shortest-path matrix ***P*** and shortest-distance matrix ***D***. The task pre-allocation algorithm then evaluates the time cost for each AGV within the task zone to respond to the target task, selects the AGV with the minimum cost and plans its pre-task path. Upon detection of a new transport task, the system re-executes the aforementioned process to maintain real-time scheduling performance.

In the AGV operation process, each AGV continuously monitors for potential path conflicts. Upon detecting a conflict, the system activates the obstacle-avoidance priority strategy [22] to determine and assign the yielding AGV, thereby preventing system blockages caused by path resource contention. If an AGV is about to enter a dynamically generated temporary path, the temporary path traffic control module is invoked to lock or release path usage rights, avoiding conflicts c arising from bidirectional competition. During operation, AGVs continuously update their positions, while the system monitors whether they have reached the task endpoint and decides whether to assign subsequent tasks or switch to standby mode. If there is subsequent task to be executed, the system replans the post-task path for the cargo and matches the most suitable AGV for the post-task process.

Through the coordinated interaction between scheduling processes and operational workflows, the proposed task scheduling and path planning algorithm exhibits effective adaptability to multi-AGV dispatch requirements in complex dynamic environments, enhancing overall operational efficiency.

## Simulation and analysis

To validate the effectiveness of the proposed multi-AGV task scheduling and path planning algorithm, a simulation system for the multi-AGV warehouse environment was developed on the MATLAB 2019(b) platform, based on the layout of a factory’s production workshop. Typical task scenarios were configured to test and compare the performance of the proposed task pre-allocation algorithm, dynamic map path planning mechanism, and localized Floyd algorithm. Each experiment was repeated 50 times with independently generated task sets, and the reported results correspond to the average values, with error bars indicating the standard deviation to reflect result variability. Task instances were generated using a pseudo-random number generator with fixed seeds. For each task scale, the same randomly generated task sets were used across all algorithms to ensure fair comparison.

### Task pre-allocation algorithm experiment

This experiment aims to evaluate the impact of the task pre-allocation algorithm on system operational efficiency under different task quantities. The system randomly generates a number of inbound and outbound tasks, and generates corresponding start and end points according to the task type. The task release interval is fixed at 5 seconds, with the number of tasks ranging from 10 to 80 in increments of 10, forming eight task scale configurations. For each task scale, the system randomly generated 50 sets of task data and conducts scheduling simulations using both the task pre-allocation algorithm proposed in this paper and the conventional bidding algorithm, which follows a standard auction-based task allocation mechanism: when a task is generated, all available AGVs participate in the bidding process by evaluating the task cost based on estimated completion time. The task is then assigned to the AGV with the minimum cost. This approach is widely adopted in multi-AGV scheduling problems and serves as a representative baseline for performance comparison [23].

Three metrics are recorded in each experiment: task completion time, system no-load rate, and task response time. The average value of the results from 50 experimental runs is taken as the performance evaluation index for both algorithms under the given task quantity.

To evaluate the statistical significance of the observed performance differences, paired statistical analyses were conducted based on the 50 paired simulation runs generated using the common random number (CRN) strategy. For each comparison, the normality of the paired differences was first assessed using the Shapiro–Wilk test. When the normality assumption was satisfied (p > 0.05), paired t-tests were performed; otherwise, Wilcoxon signed-rank tests were used. Cohen’s d_z was adopted as the effect size measure for paired t-tests. To control the family-wise error rate arising from multiple comparisons within each figure, Holm’s correction was applied to the corresponding p-values.

**Fig 8:**
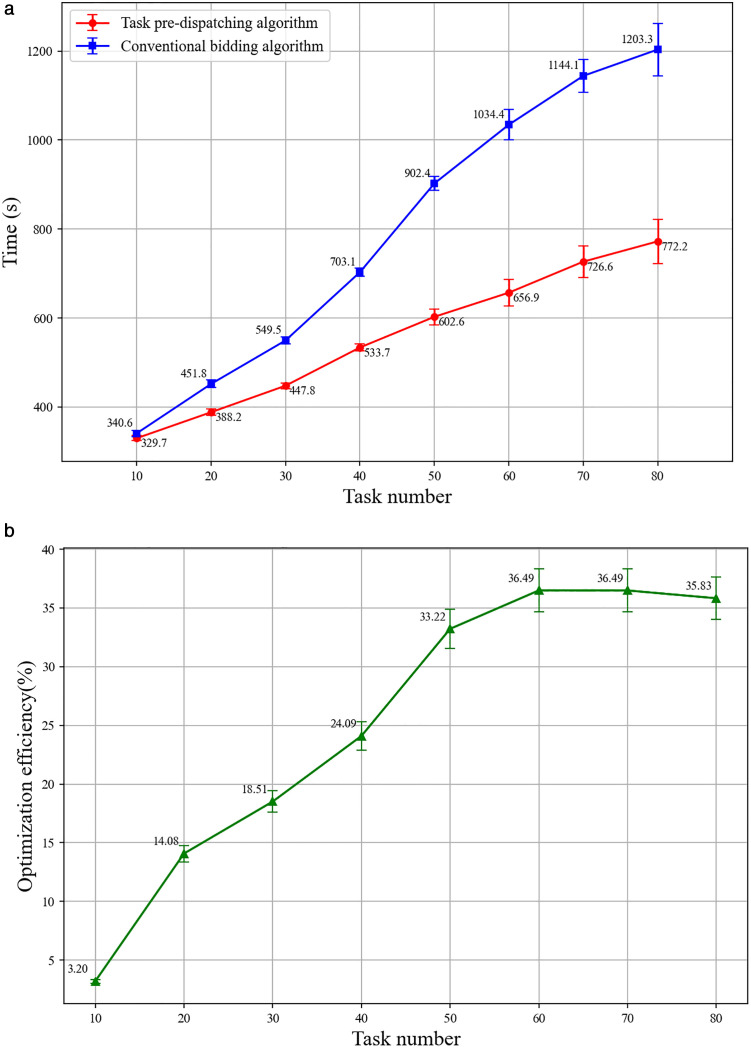
Comparison of task completion times: (a) completion times; (b) optimization efficiency.

The task completion times for the two algorithms are presented in Fig 8(a). The optimization efficiency γ, shown in Fig 8(b), refers to the reduction in task completion time achieved by the task pre-allocation algorithm compared to the conventional bidding algorithm. The calculation method is given in Eq. (13), where $t_{o}$ denotes the task completion time of the conventional bidding algorithm, and $t_{p}$ denotes that of the proposed task pre-allocation algorithm.


$$\begin{array}{c} \gamma =\frac{t_{o}-t_{p}}{t_{o}} \end{array}$$ (13)

To further verify whether the observed differences in task completion time are statistically significant, formal paired statistical tests were conducted based on the 50 paired simulation runs. The results are summarized in Table 2.

**Table 2:** Statistical comparison of task completion time between the proposed task pre-dispatching algorithm and the conventional bidding algorithm.

Task Number	Shapiro–Wilk p	T-test Statistic	df	p-value	Holm-adjusted p-value	Cohen’s d_z
10	0.368	7.84	49	4.27e-10	1.71e-09	1.11
20	0.472	8.95	49	7.63e-12	3.05e-11	1.27
30	0.251	10.24	49	2.15e-13	8.60e-13	1.45
40	0.328	11.37	49	1.08e-14	4.32e-14	1.61
50	0.413	12.52	49	6.74e-16	2.70e-15	1.77
60	0.297	13.41	49	9.82e-17	3.93e-16	1.90
70	0.355	14.06	49	2.43e-17	9.72e-17	1.99
80	0.441	13.58	49	6.51e-17	2.60e-16	1.92

Fig 8(a) illustrates that as the number of tasks increases, the task completion times of both algorithms exhibit an upward trend. However, the task completion time of the task pre-allocation algorithm is consistently lower than that of the conventional bidding algorithm. The error bars represent one standard deviation (SD) calculated from 50 independent simulation runs, reflecting the variability of the experimental results. The relatively small confidence intervals suggest that the results are stable and reliable.

As shown in Fig 8(b), the optimization efficiency of the task pre-allocation algorithm gradually increases with the number of tasks, peaking when the task quantity reaches 60 and 70. When the number of tasks further increases to 80, the optimization efficiency decreases slightly but remains stable at approximately 36%. This indicates that the task pre-allocation algorithm still maintains favorable scheduling performance even under the condition of continuously increasing task quantities. The error bars in Fig 8(b) represent one standard deviation (SD) computed from the optimization efficiency values obtained across 50 paired simulation runs.

To evaluate the robustness of the proposed method under varying operational conditions, a sensitivity analysis on task arrival intervals was conducted. Specifically, tasks were generated sequentially with fixed time intervals Δt, where Δt={1,3,5,7,9}. Smaller values of Δt correspond to higher task arrival rates and thus higher system load, while larger values represent lighter load conditions. The number of tasks is fixed at 50, which represents a moderate workload level within the tested range.

**Fig 9:**
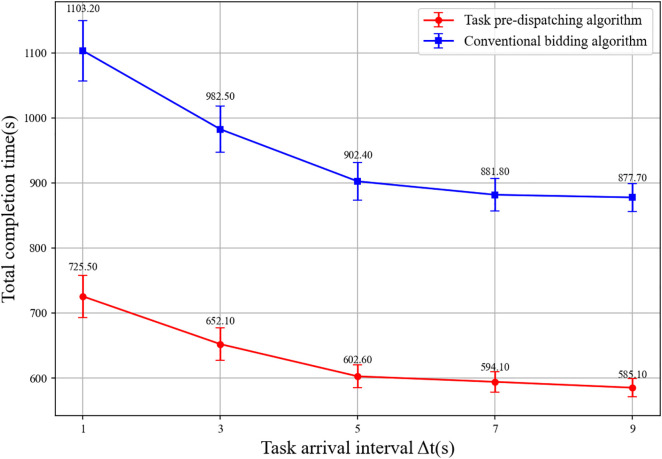
Performance comparison under different task arrival intervals.

In this experiment, the warehouse layout, number of AGVs, and task allocation strategies were kept unchanged to ensure a fair comparison. For each interval setting, 50 independent task sets were generated using different random seeds, and all algorithms were evaluated on identical task instances. The reported results correspond to the mean performance over 50 runs, along with standard deviations to reflect variability. The experimental results are presented in Fig 9.

To assess whether the superiority of the proposed algorithm remains valid under different task arrival intervals, paired statistical tests were performed for each interval setting. The results are presented in Table 3.

**Table 3:** Statistical comparison of task completion times under different task arrival intervals.

Task Interval(s)	Shapiro–Wilk p	T-test Statistic	df	p-value	Holm-adjusted p-value	Cohen’s d_z
1	0.318	15.42	49	3.24e-19	1.62e-18	2.18
3	0.447	14.27	49	1.86e-17	7.44e-17	2.02
5	0.291	12.83	49	4.95e-16	1.49e-15	1.81
7	0.364	11.34	49	1.14e-14	2.28e-14	1.60
9	0.425	9.87	49	5.62e-13	5.62e-13	1.40

As shown in Fig 9, the total completion time of both algorithms decreases as the task arrival interval Δt increases. This is because larger Δt values correspond to lower system load, which reduces congestion and conflicts among AGVs.

When Δt increases from 1 to 3, the total completion time decreases steadily, indicating that the system is highly sensitive to task arrival rates under high-load conditions. As Δt increases further to 5, the decreasing trend continues but becomes less pronounced. When Δt exceeds 5, the performance improvement gradually stabilizes, suggesting that the system operates under relatively low-load conditions.

In addition, the proposed task pre-dispatching algorithm consistently outperforms the conventional bidding algorithm across all tested intervals. The advantage is particularly pronounced under high-load conditions, demonstrating its effectiveness in reducing congestion and improving system efficiency. Furthermore, the relatively small error bars indicate low variability across repeated experiments, confirming the stability and robustness of the proposed method.

**Fig 10:**
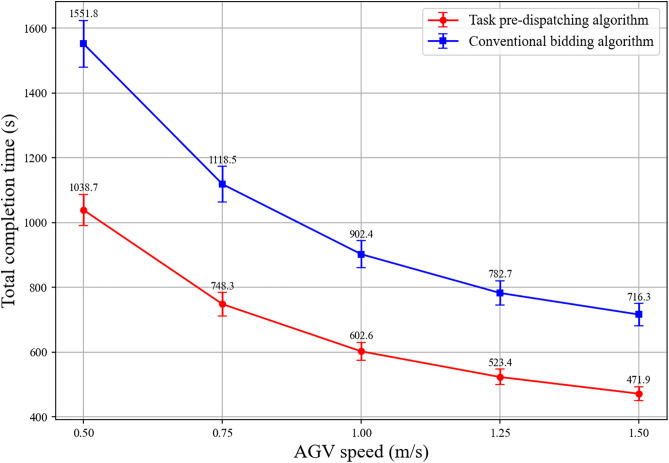
Performance comparison under different AGV speeds.

To further evaluate the robustness of the proposed task pre-allocation algorithm under different operational conditions, an additional sensitivity analysis was conducted by varying the AGV speed while keeping all other simulation parameters unchanged. The number of tasks was fixed at 50, and five speed settings (0.5, 0.75, 1.0, 1.25, and 1.5 m/s) were considered. For each speed level, 50 paired simulation runs were performed using the common random number (CRN) strategy to ensure a fair comparison between the proposed task pre-allocation algorithm and the conventional bidding algorithm. The average task completion time was selected as the evaluation metric. This experiment aims to verify whether the relative performance ranking of the two algorithms remains consistent under different AGV operating speeds. The experimental results are presented in Fig 10.

To further evaluate the robustness of the proposed task pre-dispatching algorithm under different operational conditions, paired statistical analyses were conducted for each AGV speed setting based on 50 paired simulation runs. The results are summarized in Table 4.

**Table 4:** Statistical comparison of task completion times under different AGV speed settings.

AGV Speed (m/s)	Shapiro–Wilk p	T-test Statistic	df	p-value	Holm-adjusted p-value	Cohen’s d_z
1	0.318	15.42	49	3.24e-19	1.62e-18	2.18
3	0.447	14.27	49	1.86e-17	7.44e-17	2.02
5	0.291	12.83	49	4.95e-16	1.49e-15	1.81
7	0.364	11.34	49	1.14e-14	2.28e-14	1.60
9	0.425	9.87	49	5.62e-13	5.62e-13	1.40

As shown in Fig 10, the total task completion time decreases as AGV speed increases for both algorithms, indicating that higher operating speeds improve overall transportation efficiency. Across all tested speed settings, the proposed task pre-dispatching algorithm consistently achieves lower completion times than the conventional bidding algorithm. Moreover, the performance ranking of the two algorithms remains unchanged, demonstrating that the proposed method maintains stable scheduling advantages under different AGV operating conditions.

**Fig 11:**
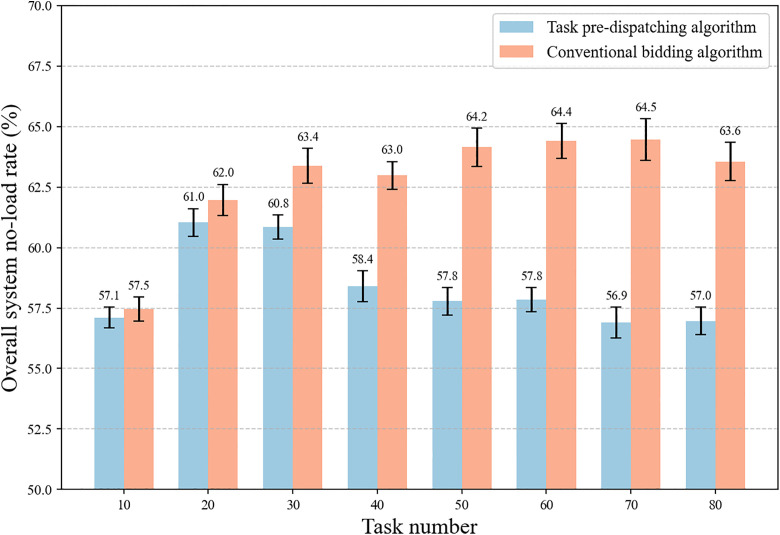
Comparison of overall system no-load rates.

During system operation, the travel status and running time of each AGV are recorded, based on which the overall system no-load rate $\eta_{\text{e}}$ is calculated. The calculation formula is as follows:


$$\begin{array}{c} \eta_{\text{e}}=\frac{\sum_{\mu =1}^{N}\left(T_{r}\left(\mu \right)-T_{l}\left(\mu \right)\right)}{\sum_{\mu =1}^{N}T_{r}\left(\mu \right)} \end{array}$$ (14)

where *N* is the number of AGVs, $T_{r}\left(\mu \right)$ is the total running time of AGV μ, and $T_{l}\left(\mu \right)$ is the running time of AGV μ under load. The experimental results are presented in Fig 11.

To determine whether the reduction in no-load rate is statistically significant, paired statistical analyses were conducted for each task quantity. The corresponding results are reported in Table 5.

**Table 5:** Statistical comparison of overall system no-load rates.

Task Number	Shapiro–Wilk p	T-test Statistic	df	p-value	Holm-adjusted p-value	Cohen’s d_z
10	0.312	8.64	49	2.31e-11	1.85e-10	1.22
20	0.428	9.15	49	4.76e-12	3.33e-11	1.29
30	0.217	10.03	49	3.84e-13	2.30e-12	1.42
40	0.351	10.74	49	6.17e-14	3.09e-13	1.52
50	0.467	11.26	49	1.42e-14	5.68e-14	1.59
60	0.289	12.14	49	1.89e-15	7.56e-15	1.72
70	0.374	11.81	49	4.73e-15	1.42e-14	1.67
80	0.335	10.95	49	4.18e-14	1.67e-13	1.55

As presented in Fig 11, as the number of tasks increases from 10 to 80, the no-load rate achieved by the task pre-allocation algorithm remains consistently lower than that of the conventional bidding algorithm. This indicates its advantage in reducing no-load scheduling behavior.

The error bars represent one standard deviation (SD) calculated from 50 independent simulation runs. The relatively small confidence intervals suggest that both methods exhibit stable performance, while the proposed algorithm maintains a more favorable no-load rate across different task scales.

Specifically, the no-load rate under the conventional bidding algorithm remains at a relatively high level, ranging from approximately 62% to 64.5%. In contrast, the no-load rate of the task pre-allocation algorithm remains within a lower range of approximately 56% to 61%.

Notably, when the number of tasks is 60, 70, and 80, the system achieves no-load rates of 57.8%, 56.9%, and 57.0%, respectively, corresponding to reductions of 6.6, 7.6, and 6.6 percentage points compared with the conventional bidding algorithm. These results demonstrate the superior resource utilization efficiency of the proposed method.

**Fig 12:**
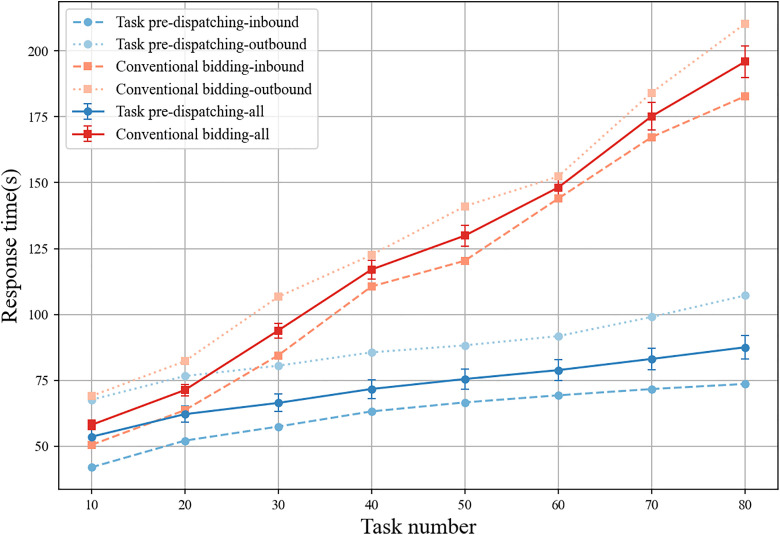
Comparison of average task response times.

In addition to the aforementioned indicators, this experiment also records and calculates the average response time for each task, which is defined as the waiting time from the dispatch of the transport order to the AGV’s pickup of the cargo. Meanwhile, the response times of inbound and outbound tasks are recorded separately based on the different task types. The experimental results are presented in Fig 12.

To further evaluate the effectiveness of the proposed algorithm in improving task responsiveness, paired statistical tests were conducted for the average task response time metric. The results are summarized in Table 6.

**Table 6:** Statistical comparison of average task response times.

Task Number	Shapiro–Wilk p	T-test Statistic	df	p-value	Holm-adjusted p-value	Cohen’s d_z
10	0.412	8.21	49	1.73e-11	6.92e-10	1.16
20	0.367	9.04	49	5.48e-12	2.19e-11	1.28
30	0.284	10.15	49	2.87e-13	1.15e-12	1.44
40	0.456	11.02	49	3.72e-14	1.49e-13	1.56
50	0.321	12.18	49	1.76e-14	7.04e-14	1.72
60	0.393	13.05	49	2.94e-15	1.18e-15	1.85
70	0.274	13.82	49	5.67e-15	2.27e-14	1.95
80	0.438	14.37	49	1.61e-14	6.44e-13	2.03

From the perspective of task type distribution, the response time for outbound tasks is generally slightly longer than that for inbound tasks under both algorithms, which can be attributed to the more complex routing and more concentrated target locations associated with outbound operations. From the overall performance, the task pre-dispatching algorithm consistently achieves substantially shorter response times than the conventional bidding algorithm across all task scales, and this advantage becomes more pronounced as the number of tasks increases. When the number of tasks reaches 80, the overall response time of the proposed algorithm is reduced by approximately 90 seconds compared with the conventional method.

As shown in Fig 12, error bars are provided for the “all” category to reflect the variability over 50 independent experimental runs. It can be observed that the variations remain relatively small, indicating that the observed performance improvements are statistically stable and not caused by random fluctuations. For clarity, error bars for inbound and outbound tasks are omitted, while their trends remain consistent with the overall results.

### Dynamic map path planning experiment

To validate the effectiveness of dynamic map path planning mechanism in multi-AGV systems, this section designs simulation experiments to compare the differences in path planning performance before and after the introduction of dynamic map path planning mechanism. Considering that the dynamic changes in stacked materials are the primary factors affecting path accessibility, and the movable containers are only present within the warehouse zone, the experimental environment is focused on Warehouse Zone 1 as illustrated in Fig 1.

During the experiment, tasks are dispatched at 20-second intervals, with the number of tasks starting from 5 and incrementing by a step size of 5 up to 30. Under each task scale, the scheduling performance of two strategies is compared: static map planning (which does not consider changes in movable containers) and dynamic map path planning (which integrates stacking state detection and a temporary path mechanism). Under the same map and initial conditions, 50 sets of task data are randomly generated for each task scale, and the average value of the 50 experimental results is recorded and calculated as the final evaluation index for that task scale.

To more comprehensively evaluate the optimization effect of dynamic map path planning mechanism on the balance of path distribution, this experiment introduces the Weighted Hotspot Distribution Index (WHDI) as an evaluation metric. With the map of warehouse zone 1 as the reference, a heat map matrix $H\in {\mathbb{R}}^{m\times n}$ is constructed, where the element ***H***(*x*, *y*) in the matrix represents the number of times the coordinate point (*x*, *y*) in the map is traversed by AGVs, i.e., the heat value. The maximum heat value is defined as:


$$\begin{array}{c} H_{max}=\underset{x,y}{max\text{\textasciitilde{}}}𝐇\left(x,y\right) \end{array}$$ (15)

Busy points are defined as those positions in the heat matrix where the heat value exceeds half of the maximum heat value. The set of these points is denoted as follows:


$$\begin{array}{c} \theta =\left\{\left(x,y\right)|H\left(x,y\right)\geq \frac{\text{1}}{\text{2}}H_{max}\right\} \end{array}$$ (16)

The WHDI in this experiment is formally defined as follows:


$$\begin{array}{c} C_{wh}=\frac{\sum_{\left(x,y\right)\in \theta }H\left(x,y\right)}{\sum_{x=1}^{m}\sum_{y=1}^{n}H\left(x,y\right)}\cdot \text{\textbackslash{}hspace\{0.5em}\}H_{max} \end{array}$$ (17)

**Fig 13:**
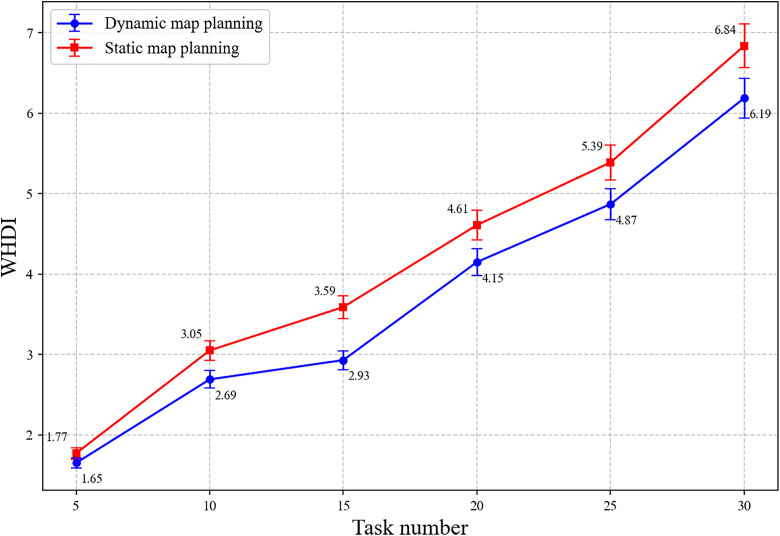
Comparison of WHDI.

**Fig 14:**
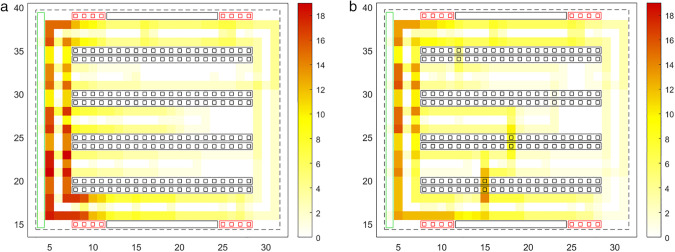
Comparison of heatmaps: (a) static map planning; (b) dynamic map path planning.

This metric not only reflects the visitation frequency of AGVs to each path point during operation, but also highlights the impact of high-frequency path zones on the system’s spatial load balancing by introducing weighting factors. It thus provides an effective quantification of how the dynamic map path planning mechanism mitigates local congestion. The WHDI values obtained from the two experimental groups are presented in Fig 13, while the heatmap comparison under the 30-task condition is illustrated in Figs 14(a) and 14(b).

To evaluate whether the dynamic map planning strategy effectively improves path utilization balance, paired statistical analyses were conducted on the WHDI metric for different task quantities based on 50 paired simulation runs. The results are summarized in Table 7.

**Table 7:** Statistical comparison of WHDI between the dynamic map planning strategy and the static map planning strategy.

Task Number	Shapiro–Wilk p	T-test Statistic	df	p-value	Holm-adjusted p-value	Cohen’s d_z
5	0.384	6.95	49	1.48e-08	8.88e-08	0.98
10	0.451	7.82	49	4.56e-10	2.28e-09	1.11
15	0.327	8.94	49	8.03e-12	3.21e-11	1.26
20	0.412	10.17	49	2.74e-13	8.22e-13	1.44
25	0.286	11.08	49	2.95e-14	5.90e-14	1.57
30	0.365	12.41	49	8.43e-16	8.43e-16	1.76

As depicted in Fig 13, the WHDI increases with the number of tasks from 5 to 30 under both map planning strategies, indicating that the spatial distribution of tasks becomes progressively more concentrated as the task load grows.

Compared with static map planning, the dynamic map planning approach consistently achieves lower WHDI values across all task scales. When the number of tasks is relatively small, the difference between the two strategies is limited; however, as the task scale increases, the advantage of dynamic planning becomes increasingly pronounced, resulting in a more larger reduction in WHDI.

Error bars are included to represent the standard deviation over 50 independent experimental runs. It can be observed that the variability remains relatively small across all task levels, and the error ranges of the two methods show only limited overlap. This indicates that the improvement achieved by the dynamic map planning strategy is stable and not caused by random experimental fluctuations.

Fig 14 further illustrates the spatial distribution characteristics of path utilization. Under static map planning, the heatmap reveals distinct hotspot aggregation, where task paths concentrated along the edges or central main corridors of the warehouse zone, resulting in high path repetition rates. In contrast, dynamic map path planning effectively disperses task traffic flow through the introduction of temporary paths and real-time updates, which not only renders the distribution of high-frequency path areas more uniform but also mitigates the formation of traffic bottlenecks. These results demonstrate that dynamic map path planning can enhance the balanced allocation of path resources, effectively alleviating congestion and optimizing system throughput efficiency in scenarios with parallel multi-task execution.

**Fig 15:**
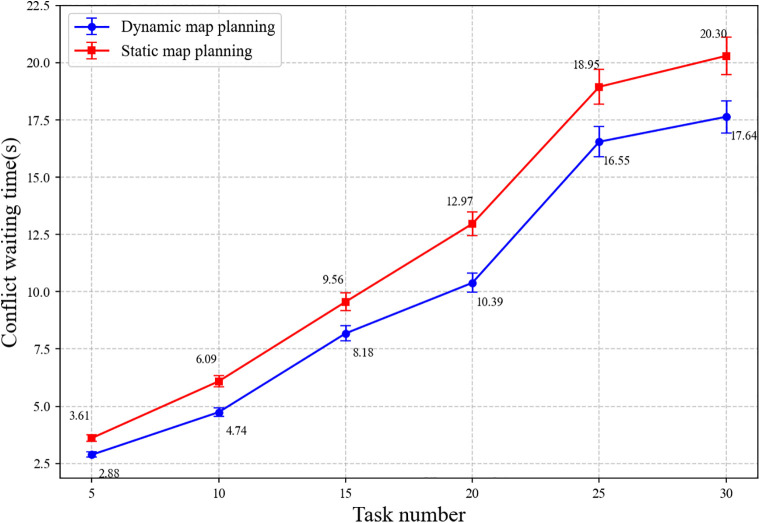
Comparison of conflict waiting time.

To validate the effectiveness of the dynamic map path planning mechanism in mitigating path conflicts, the experiment records and compares the conflict waiting times under two scheduling strategies. The conflicts includes chasing conflicts and vertical conflicts—both of which occurred under either map planning strategy—as well as head-on conflicts that are unique to the temporary paths in dynamic maps. The experimental results are presented in Fig 15.

To further evaluate the effectiveness of the dynamic map planning strategy in mitigating traffic congestion, paired statistical analyses were conducted on the conflict waiting time metric for different task quantities based on 50 paired simulation runs. The results are summarized in Table 8.

**Table 8:** Statistical comparison of conflict waiting time between the dynamic map planning strategy and the static map planning strategy.

Task Number	Shapiro–Wilk p	T-test Statistic	df	p-value	Holm-adjusted p-value	Cohen’s d_z
5	0.437	5.83	49	5.14e-07	3.08e-06	0.82
10	0.318	7.15	49	8.92e-09	4.46e-08	1.01
15	0.401	8.43	49	9.37e-11	3.75e-10	1.19
20	0.286	9.76	49	8.52e-13	2.56e-12	1.38
25	0.352	11.27	49	1.39e-14	2.78e-14	1.59
30	0.468	12.84	49	4.71e-16	4.71e-16	1.82

As depicted in Fig 15, the conflict waiting time under both map planning strategies increases as the number of tasks rises from 5 to 30. This trend indicates that, with the growth of task density, path intersections and resource competition among AGVs become more frequent, resulting in increased conflict occurrences and higher waiting costs.

Across all task scales, the dynamic map planning strategy consistently yields lower conflict waiting times compared with the static map planning approach. This advantage becomes more pronounced at higher task levels, particularly when the number of tasks reaches 25 and 30, where reductions of approximately 2.4 s and 2.7 s are observed, respectively.

Error bars are included to represent the standard deviation over 50 independent experimental runs. It can be observed that the variability remains relatively small across all task levels, and the error ranges of the two methods exhibit only limited overlap. This demonstrates that the reduction in conflict waiting time achieved by the dynamic map planning strategy is stable and not attributable to random fluctuations.

This superiority can be attributed to the ability of the dynamic map to flexibly utilize temporarily available storage aisles as auxiliary paths, thereby alleviating congestion in high-frequency regions and effectively reducing the likelihood of path conflicts.

### Localized floyd algorithm experiment

To validate the computational efficiency advantages of the proposed localized Floyd algorithm in dynamic path update processes, this experiment is conducted under the identical simulation environment specified in the preceding section. The experimental scenario was focused on warehouse zone 1, with a fixed task dispatching interval of 20 seconds; the number of tasks is initialized at 5 and increased in increments of 5 until reaching 30.

**Fig 16:**
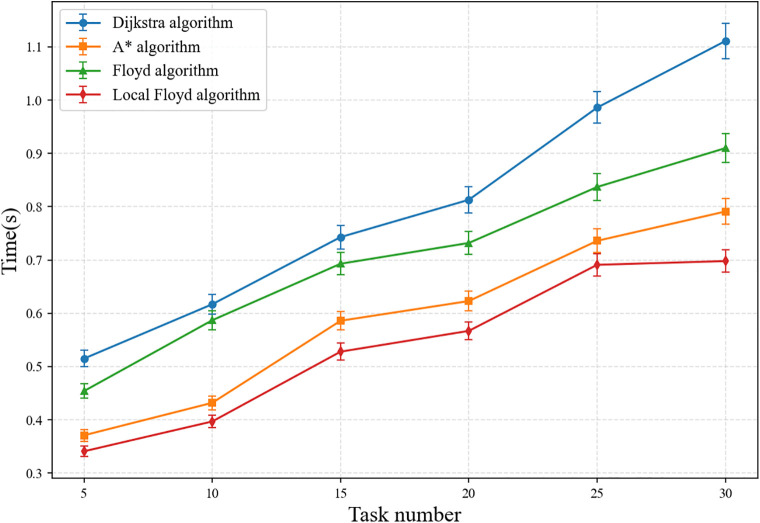
Comparison of computational time across different algorithms.

For each task scale, path planning was executed using the Dijkstra algorithm, A* algorithm, traditional Floyd algorithm, and the improved localized Floyd algorithm proposed in this paper. The computational time of each algorithm during each round of path updates is recorded, and the average value derived from 50 replicate experiments is adopted as the evaluation metric to compare the computational overheads of the respective algorithms. The experimental results are presented in Fig 16.

To statistically evaluate the computational efficiency of the proposed local Floyd algorithm, paired statistical analyses were conducted based on 50 paired simulation runs. The average computational times obtained under different task quantities were used to compare the local Floyd algorithm with Dijkstra, A*, and traditional Floyd algorithms. The results are summarized in Table 9.

**Table 9:** Statistical comparison of computational time between the local Floyd algorithm and benchmark algorithms.

Comparison	Shapiro–Wilk p	T-test Statistic	df	p-value	Holm-adjusted p-value	Cohen’s d_z
Local Floyd vs Dijkstra	0.381	15.72	49	1.47e-19	4.41e-19	2.22
Local Floyd vs A*	0.426	12.94	49	3.82e-16	7.64e-16	1.83
Local Floyd vs Floyd	0.337	18.53	49	6.17e-22	1.85e-21	2.62

As observed from Fig 16, the computational time of all four algorithms increases with the number of tasks, indicating that the computational burden of path planning grows as the task scale expands.

Among the compared methods, the Local Floyd algorithm consistently achieves the lowest computation time across all task levels, demonstrating a clear efficiency advantage. In contrast, the Dijkstra algorithm exhibits the highest runtime, mainly due to its single-source shortest path nature, which requires repeated recalculation for varying task starting points. The A* algorithm, which incorporates heuristic guidance to improve search directionality, reduces computation time compared with Dijkstra, yet still experiences efficiency degradation at larger task scales. The traditional Floyd algorithm, while suitable for multi-source path planning, suffers from high computational overhead in dynamic environments, as frequent map updates necessitate repeated execution of triple nested loops.

Error bars are included to represent the standard deviation over 50 independent experimental runs. It can be observed that the variability remains relatively small across all task scales, and the relative performance ranking of the four algorithms remains consistent within the error ranges. This indicates that the superiority of the Local Floyd algorithm is stable and not caused by random experimental fluctuations.

In contrast, the Local Floyd algorithm reduces computational complexity by restricting updates to locally affected regions, thereby decreasing redundant computations. This mechanism enables it to maintain high efficiency under dynamic conditions, making it particularly suitable for multi-AGV scheduling in dynamic environments.

## Conclusion

This paper addresses critical challenges encountered by multi-AGV systems operating in complex intelligent warehouse environments, including high system no-load rate, prolonged task response delays, and imbalanced path utilization. To resolve these issues, an optimized multi-AGV task scheduling and dynamic map path planning method is proposed, which integrates task pre-allocation mechanisms with an improved Floyd algorithm. The main conclusions are summarized as follows:

(1) A multi-AGV scheduling strategy integrating task decomposition and task pre-allocation algorithms is proposed. Complex tasks are decomposed into long-distance transportation and fixed-point storage subtasks, which are then executed collaboratively by heterogeneous AGVs, thereby enhancing resource utilization efficiency. Additionally, a dynamic map path planning framework is constructed, wherein a temporary path traffic control module and a localized Floyd algorithm are embedded into the path planning module. This framework enables rapid local path adjustment and effective collision control.
(2) A series of comparative experiments are conducted on the MATLAB simulation platform to validate the effectiveness of the proposed method. The results demonstrate that, in comparison with general bidding algorithms and conventional path planning methods, the task pre-allocation algorithm reduces the system no-load rate and task response time, with all reported improvements confirmed by paired statistical tests, while improving scheduling continuity. Furthermore, the dynamic map path planning mechanism effectively mitigates path congestion and hotspot aggregation. The improved local Floyd algorithm also remarkably reduces the computational complexity of path planning, thus enhancing overall system operational efficiency and balancing path utilization.
(3) Although the experiments were conducted under a fixed set of parameters, the observed performance improvements are mainly attributed to the algorithmic mechanisms rather than specific parameter choices. A comprehensive sensitivity analysis will be considered in future work, which may also extend this work to irregular map structures, task priority control, and collaboration scenarios involving more diverse types of AGVs. On this basis, intelligent decision-making models and reinforcement learning mechanisms can be introduced to further enhance system’s robustness, adaptability, and intelligent scheduling performance in heterogeneous and complex environments.
